# Non Puerperal Uterine Inversion Due to Submucous Myoma: A Case Report 

**Published:** 2018-09

**Authors:** Salmeh Dadgar, Seyedeh Azam Pourhosseini

**Affiliations:** Department of Obstetrics and Gynecology, Faculty of Medicine, Mashhad University of Medical Sciences, Mashhad, Iran

**Keywords:** Leiomyoma, Uterine Inversion, Submucusal Myoma

## Abstract

Spontaneous uterine inversion rarely occurs in times other than the postpartum period. This condition is usually associated with the presence of a polypoid mass in the fundus, which is often a uterine leiomyoma or, in rare cases, a uterine sarcoma. Herein we report a case of a multipara, 51-year-old woman, presented with pelvic pain and vaginal bleeding. On the speculum examination, a circular mass of about 10 Cm, was observed in the vagina. In an ultrasound, a 55×62 mm intramural fibroid was observed. the patient was scheduled for surgery. After accessing the abdominal cavity, the patient was diagnosed with the uterine inversion. A longitudinal incision was made on the retraction ring. The base of the prolapsed mass was clamped at the incision site, and the mass was driven up into the vaginal canal and a total hysterectomy was performed.

## Introduction

Uterine inversion is a condition in which the fundus collapses into the endometrial cavity, which can turn the uterus partially or completely inside out (1). Spontaneous uterine inversion rarely occurs in times other than the postpartum period and only includes 5% of the total cases of uterine inversion. This condition is usually associated with the presence of a polypoid mass in the fundus, which is often a uterine leiomyoma or, in rare cases, a uterine sarcoma (2).

On the other hand, the non-puerperal uterine inversion mainly occurs around the age of menopause and is often difficult to be clinically diagnosed. There must be a strong clinical suspicion to diagnose this condition in cases of prolapsed myoma, absence of uterus in its normal position in the bimanual examination, and palpation of concavity on the surface of the uterus(3).

The clinical symptoms of non-puerperal uterine inversion could differ based on the acute or chronic state of the disease. In acute uterine inversion, the main symptoms are extreme pain and vaginal bleeding. On the other hand, the major manifestations of the chronic type of this disease are pelvic pain, vaginal discharge, irregular uterine bleeding, and anemia (4).

Clinical examination might help in the diagnosis of uterine inversion, in which the mass bulged out from the vagina is examined and the probe test is negative. Additionally, in the ultrasound, which reveals the fundal area, a U-shaped depressed longitudinal groove from the uterine fundus to the center of the inversion shows the uterine inversion. Furthermore, the ultrasound images may show the “target sign”, which indicates the presence of fluid in the space between the inverted uterine and vaginal wall (5).

This condition can be treated using surgical methods including abdominal or vaginal techniques. The Spinelli and Kustner techniques are used for vaginal surgery, and the Huntington and Haultaim procedures are the abdominal techniques. However, if the non-puerperal uterine inversion is caused by a mass, first, the mass must be removed, and then the uterine inversion be corrected (6).

## Case report

Herein, we reported a case of a 51-year-old woman (parity: 3, labor: 2, delivery: 1), presented with pelvic pain and vaginal bleeding. The pelvic pain was initiated two days before admission and was intensified the morning of hospitalization. The patient had a three-year history of menometrorrhagia, for which she had undergone a diagnostic curettage on August 15, 2016, the pathology of which was reported as "inactive endometrium and endocervical polyp".

Upon admission, the patient’s hemorrhage was similar to menstrual bleeding. In the physical examination, the abdomen was soft with no obvious tenderness. However, on the speculum examination, a circular mass of about 10 cm, similar to a pediculated myoma, was observed in the vagina, extended to the entrance of the vagina during Valsalva maneuver.

Bimanual examination also revealed the occupation of the vaginal space by the mentioned mass, the thick base of which could be touched. However, the uterus was impalpable, and the hemorrhage was analogous to the menstrual bleeding. In an ultrasound performed on April 23, 2016, the uterine size was 56×79×109 mm, and a 55×62 mm intramural fibroid was observed in the posterior wall of the uterus, which pressurized the adjacent endometrium. Moreover, the endometrial thickness was reported to be 7 mm, and the adnexa were normal.

The vital signs were stable on admission; however, the patient looked pale. The patient had a hematocrit level of 24% and a hemoglobin level of 7 mg/dL; however, other tests were normal. To improve the patients' anemia, two units of packed cell were transfused, and the patient was scheduled for surgery.

**Figure 1:**
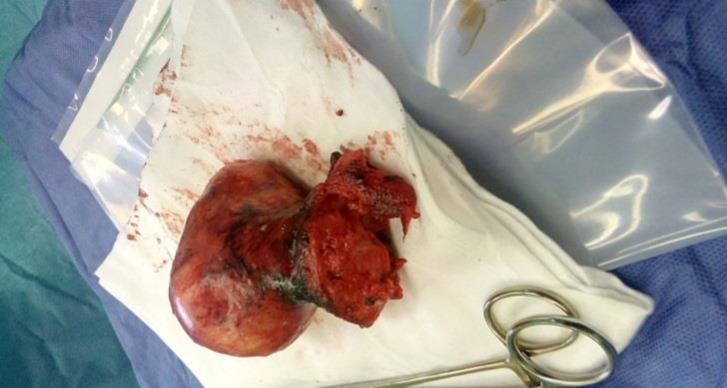
Uterine inversion after opening the fascia

In the operation room, the patient was put in a dorsal lithotomy position and examined under anesthesia. Due to the lack of access to basic myoma, the patient underwent an abdominal hysterectomy. Her abdomen was opened with a median incision in the area of the previous scar. After opening the fascia and peritoneum and accessing the abdominal cavity, the patient was diagnosed with the uterine inversion based on detecting utero-ovarian round ligament and not observing the fondus (figure1). 

At first, it was attempted to treat the inversion through applying tension on the ligament, which was not successful due to the extent of the inversion. Therefore, the utero-ovarian round ligaments were ligated on both sides, and then a longitudinal incision was made on the retraction ring after the lowering of the bladder to reduce the inversion; however, it was not successful. The base of the prolapsed mass was clamped at the incision site, and the mass was driven up into the vaginal canal. Subsequently, the mass was removed by the assistant using tenaculum.

Afterwards, the uterine arteries were ligated on both sides, and a total hysterectomy was performed after obtaining the cardinal and uterosacral ligaments. Subsequently, the myoma and uterus were sent to the laboratory for pathological examination.

## Discussion

Uterine inversion, which might be partial or complete, is caused by the collapse of the fundus into the endometrial cavity (1). This is a rare condition, which might occur after vaginal delivery or cesarean section. However, it is regarded as a life-threatening obstetrical emergency for mothers; as a result, it needs immediate diagnosis and treatment(1). The uterine inversion after childbirth could be associated with uterine atony and excessive postpartum hemorrhage, leading to maternal shock and mortality (1).

The occurrence of spontaneous uterine inversion in any time other than postpartum period is significantly rare and accounts for only 5% of all cases of uterine inversions. This condition is usually associated with the presence of a polypoid mass in the fundus, which is often a uterine leiomyoma or, in rare cases, a uterine sarcoma (2).

There are four types of uterine inversions based on the extent of this condition (7): 

First degree: incomplete inversion, where the fundus is inside the endometrial cavity. 

Second degree: complete inversion, where the fundus protrudes through the cervical canal.

Third degree: prolapsed inversion, where the fundus protrudes to the vaginal outlet or prolapses beyond it.

Fourth degree: total inversion, where the vagina and the uterus are both inverted.

In terms of time, uterine inversions are divided into three categories (8): 

Acute: the uterine inversion occurs over the first 24 h of a vaginal delivery.

Subacute: the uterine inversion happens between 24 h and four weeks after a vaginal delivery.

Chronic: the uterine inversion takes place after more than one month following a vaginal delivery.

The non-puerperal uterine inversion mostly occurs around the time of menopause and its clinical diagnosis is difficult. In cases with prolapsed myoma, absence of uterus in its normal position in bimanual examination, or palpation of a concavity on the surface of the uterus, there must be a strong clinical suspicion to diagnose this condition(3).

The clinical symptoms of non-puerperal uterine inversion differ based on the acute or chronic form of the disease. In the acute inversion, the major symptoms are extreme pain and vaginal hemorrhage. On the other hand, the main manifestations of the chronic inversion are pelvic pain, vaginal discharge, irregular vaginal hemorrhage, and anemia (4).

A clinical examination might help detect a uterine inversion, through which the mass protruded from the vagina is examined, and the probe test is negative. In the ultrasound, which reveals the fundal area, a U-shaped depressed longitudinal groove from the uterine fundus to the center of the inversion shows the uterine inversion. Furthermore, the ultrasound images may show the “target sign”, which indicates the presence of fluid in the space between the inverted uterine and vaginal wall (5).

The treatment of the uterine positions in non-puerperal uterine inversion could be different based on the type of the inversion (i.e., acute and chronic). In the acute form of this disease, it is possible to manually restore the uterus to its normal position. However, in the chronic type, the manual repositioning is not possible, and it requires surgery, which will be planned based on the age of the patient and the patient’s decision on uterine repositioning or hysterectomy (6).

This condition can be treated using abdominal or vaginal surgical methods. In the Spinelli technique, the incision is made on the anterior part of the contracted cervical ring, and the bladder dissection will occur if needed. On the other hand, in the Kustner method, the incision is made on the posterior uterine wall to ensure having sufficient space to perform the uterine reduction process (6).

There are two abdominal surgery methods for the treatment of uterine inversion including Huntignton and Haultaim procedures. In the former method, first, the ring area is dilated using the fingers, and then there will be a gentle upward traction on the round ligament. On the other hand, in the Haultaim method, at first, a vertical incision is made on the posterior part of the ring, and then a gentle tension is applied on the ligament in order to restore the uterine inversion. However, if the non-puerperal uterine inversion is caused by a mass, first the mass must be removed, and then the uterine inversion is managed (9).

In a similar case study that was carried out in 2013, it was reported that a 65-year-old woman was a candidate for total vaginal hysterectomy due to vaginal prolapse. However, given the presence of uterine inversion, the case initially underwent a modified Kustner surgery, during which the pouch of Douglas was opened via a vertical incision on the posterior vaginal wall. Afterwards, a longitudinal incision was made on the uterus to manage the inversion, and then, a vaginal hysterectomy was performed (10).

In another similar case report, a 51-year-old woman presented with uterine inversion caused by submucosal myoma. After performing vaginal myomectomy, the sample was analyzed via cytopathology and found to be benign. After two weeks, the case underwent a laparotomy with total abdominal hysterectomy, the results of which were indicative of uterine inversion. Subsequently, a modified Haultain procedure was perform, followed by colpoperineorrhaphy (11).

In a report from a clinic in Karachi, Pakistan, a 39-year-old woman presented with long-term vaginal hemorrhage, impaired consciousness, and hemoglobin level of 4.5 mg/dL. Immediate anemia correction was performed on the patient. In addition, a 7×6 cm bulky mass with bad odor bulged out of the vagina. At first, vaginal myomectomy was carried out, followed by the opening of the abdomen through a Pfannenstiel incision. Uterine inversion was completely diagnosed, and hysterectomy was performed after the uterine inversion correction (12).

## Conclusion

The non-puerperal uterine inversion mostly occurs around the time of menopause and its clinical diagnosis is difficult. The treatment of the uterine positions in non-puerperal uterine inversion could be different based on the type of the inversion (i.e., acute and chronic). In the acute form of this disease, it is possible to manually restore the uterus to its normal position. However, in the chronic type, the manual repositioning is not possible, and it requires surgery.
